# Encapsulation of D-limonene in *Lepidium perfoliatum* seed gum/PVA electrospun nanofibers: Physicochemical characterization and modeling the kinetics of release

**DOI:** 10.1016/j.crfs.2024.100966

**Published:** 2024-12-24

**Authors:** Roya Kamalpour, Arash Koocheki, Behrouz Ghorani

**Affiliations:** a Department of Food Science and Technology, Ferdowsi University of Mashhad, Mashhad, Iran; b Department of Food Nanotechnology, Research Institute of Food Science and Technology (RIFST), Mashhad, Iran

**Keywords:** D-limonene, Nanofibers, Electrospinning, *Lepidium perfoliatum* seed gum, Encapsulation, Release kinetic

## Abstract

To improve the stability of D-limonene, a protective barrier is essential to prevent degradation and maintain its integrity. Therefore, the potential of using *Lepidium perfoliatum* seed gum (LPSG) as a novel source for creating electrospun nanofibers for D-limonene encapsulation was investigated by varying LPSG concentrations (0.25%, 0.5%, 0.75%, and 1% w/v) and LPSG/PVA (Polyvinyl alcohol) mixing ratios (ranging from 100:0 to 0:100 v/v). Surface tension, electrical conductivity, zeta potential, and viscosity of solutions increased as LPSG concentration and its ratio in the LPSG/PVA blend increased. Uniform, smooth, and small size nanofibers were created by electrospinning a LPSG to PVA ratio of 30:70 (v/v) using LPSG concentrations of 0.5% (w/v) and 0.75% (w/v). The FTIR analysis demonstrated that D-limonene was physically trapped within the nanofibers and confirmed the compatibility of LPSG and PVA. Following its encapsulation inside LPSG/PVA nanofibers, D-limonene's thermal stability increased. The highest D-limonene encapsulation efficiency was 96.23% for 0.75% LPSG/PVA nanofibers, which was chosen to measure the D-limonene release kinetics in simulated food models. D-limonene was most readily released in distilled water with an explosive release mechanism. The mechanism of D-limonene release from LPSG/PVA electrospun nanofibers was best described by the Peppas-Sahlin model, and the release followed Fickian diffusion mechanism. The results of this study confirmed the potential of LPSG/PVA electrospun nanofibers to effectively trap D-limonene and improve its thermal stability.

## Highlights

• Effective encapsulation of D-limonene was achieved using LPSG/PVA electrospun nanofibers.
• Encapsulating D-limonene in nanofibers improved its thermal stability.
• The rate of D-limonene release in the distilled water was high.
• D-limonene release from nanofibers followed a non-Fickian diffusion mechanism.

## Graphical abstract

**Figure:**
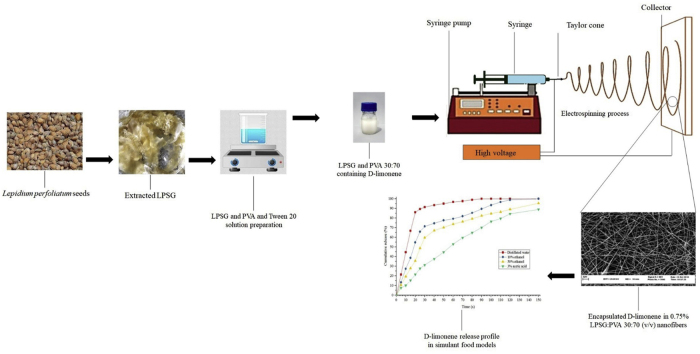

## 1 Introduction

Limonene is one of the most common phenolic compounds in citrus fruits, which play significant role in creating aroma, in addition to their health benefits (Akhavan-Mahdavi et al., 2022). D-limonene, also known as 4-isopropenyl-1-methylcyclohexene (C_10_H_16_), is a volatile and chemically unstable compound which is susceptible to degradation by heat, oxygen, and light (Francisco et al., 2022). Additionally, D-limonene is hydrophobic, which limits both its use in industry and its bioavailability (Ibáñez et al., 2020; Lopresto et al., 2019). To prevent the aforementioned issues and off flavors formation while storing D-limonene in ambient conditions, a protective barrier must be used (Chen et al., 2013; Zahi et al., 2015). It appears that encapsulation is an appropriate strategy to overcome these challenges and enable the application of D-limonene in food systems. D-limonene has so far been encapsulated using a variety of techniques, some of which include spray-drying (Castel et al., 2023), freeze-drying (Evageliou and Saliari, 2017), complex coacervation (Jun-xia et al., 2011), inclusion complex with β-cyclodextrin (Mallardo et al., 2016), oil in water nanoemulsion (Sohan et al., 2023), nanocomplexes (Ghasemi et al., 2018), pickering emulsion nanoparticles (Alehosseini et al., 2021), liposome nanoparticles (Umagiliyage et al., 2017), hydrogel (Jaafar and Thatchinamoorthi, 2018), organogel (Zahi et al., 2015), solid lipid nanoparticles (Souto et al., 2020), nano lipid particles (Yang et al., 2020), electrospray nanocapsulation (Khoshakhlagh et al., 2017), electrospun nanofibers (Lan et al., 2019).

When compared to traditional methods, electrospinning has a number of advantages for encapsulation of bioactive ingredients, including the ability to be carried out at room temperature, simplicity of use, high loading capacity and encapsulation efficiency, low-cost, and versatility (Pires et al., 2023). Electrospinning technique can be used for encapsulation of D-limonene, since it do not use high temperatures, thus protecting the volatile bioactive compounds (Akhavan-Mahdavi et al., 2022). The nanofibers formed during the electrospinning process have unique characteristics like high surface area, flexibility and porosity with remarkable stability in ambient conditions (Muthukrishnan, 2022). Three different factors, including solution properties (viscosity, surface tension, electrical conductivity, concentration, molecular weight, type of solvent), process parameters (voltage, flow rate, distance between needle's tip to collector), and ambient conditions (temperature and moisture) influence the electrospinning process (Guo et al., 2022).

Since the stability of the selected encapsulants determines the effectiveness of encapsulation, the choice of coating materials used determines the overall success of the encapsulation (Premjit et al., 2022). Polysaccharides are more desirable to be used as a wall material, due to their higher thermal resistance because they are less susceptible to some thermal challenges, such as the denaturation of protein groups and melting problems in lipids (Fathi et al., 2012; Wen et al., 2017). Different natural polysaccharides derived from plants, seaweeds, and microorganisms have been used as nanocarriers in electrospinning processes (Khoshakhlagh et al., 2018; Mendes et al., 2017; Syed et al., 2023; Zhang et al., 2022). Due to their ability to spin, polysaccharides can be classified into three groups: those that produce fibers, those that create jets, and those that cannot spin. Polysaccharide processing during electrospinning is influenced by their concentration, chemical structure, and shear thinning behavior (Mendes et al., 2017). According to Stijnman et al. (2011), there are at least two requirements that polysaccharide solutions must meet in order to be suitable for fiber formation by electrospinning: weak shear thinning behavior, and high degree of their chain entanglements.

*Lepidium perfoliatum* seed gum (LPSG), a likely anionic polysaccharide of the arabinoxylan type (Koocheki et al., 2022), could be used as a suitable viscosity modifier, texture modifier, and emulsion/foam stabilizer in the food industry due to its high viscosity, yield stress, and strong shear thinning properties (Hesarinejad et al., 2014; Koocheki et al., 2013; Mohammadkhani et al., 2024; Sadeghi et al., 2021; Soleimanpour et al., 2013a, 2013b). LPSG has been used as a wall material for encapsulation of heat sensitive or bioactive compounds (Dehghan et al., 2020; Jamshidi et al., 2020; Rezaei Savadkouhi et al., 2020). According to Jamshidi et al. (2020), the high viscosity of LPSG decreases the migration of oil to the surface, enhancing the encapsulation efficiency. This characteristic appear to enable the creation of LPSG capsules through electrospinning because high viscosity promotes the formation of the capsules through polymer chain entanglements (Khoshakhlagh et al., 2017). However, LPSG's polyelectrolyte nature, tendency to form gel at low concentrations, high electrical conductivity, and high surface tension are the main reasons for its poor electrospinnability. Addition of a non-toxic, water-soluble, biocompatible synthetic polymer, like Poly(vinyl alcohol) (PVA), can help overcome this drawback since it can reduce the repulsive forces in the charged biopolymer solutions and allow spinning of fibers (Santos et al., 2014).

According to Premjit et al. (2022), a more promising technique for increasing the efficiency of flavor encapsulation appears to be nanoencapsulation using electrospraying and electrospinning. Although LPSG was used for protecting heat-sensitive compounds, its application in the production of electrospun nanofibers for the encapsulation of sensitive compounds have not yet been investigated. Furthermore, encapsulation of D-limonene in LPSG has not been reported in the literatures before. Therefore, the main aim of this research was to fabricate LPSG electrospun nanofibers for D-limonene encapsulation. The morphology, possible interactions between the components, and thermal analysis of nanofibers were also examined, as well as the kinetics of D-limonene release in simulated food models. This study provides novel insights into the encapsulation of D-limonene within electrospun nanofibers using the advantage of LPSG to improve encapsulation efficiency through polymer chain entanglements.

## 2 Materials and methods

### 2.1 Materials

The *Lepidium perfoliatum* seeds were obtained from a local medical plant market (Mashhad, Iran). Food-grade PVA (Mw = 77000-79000 Da, 98% hydrolyzed), and D-limonene with 97% purity purchased from Sigma-Aldrich (St. Louis, MO, USA). Ethanol with a purity of 99% was provided from Scharlau Chemie (Barcelona, Spain). Other chemicals applied in this study including acetic acid, Tween-20 (HLB = 16.7), and dialysis bag (Mw = 12 Da), were obtained from Merc Co. (Darmstadt, Germany).

### 2.2 Solutions preparation

LPSG was extracted, purified and dried using the procedure provided by Koocheki et al. (2009b). The gum powder was then stored in sealed polyethylene bags at room temperature for further experiments. Different concentrations of LPSG were dissolved in distilled water (0.25%, 0.5%, 0.75%, and 1% (w/v)(and kept overnight at 4 °C for complete hydration (Monjazeb Marvdashti et al., 2017). Separately, 10% (w/v) PVA solution was made by dissolving 1g of PVA in 10 ml of deionized water, stirring continuously for 4 h at 80 °C, and then hydrating the solution overnight at 4 °C. To prepare the LPSG/PVA solutions for electrospinning, LPSG dispersions with different concentrations were mixed with PVA solution in mixing volume ratios of 100:0, 90:10, 70:30, 50:50, 30:70, 10:90, and 0:100.

### 2.3 Solutions properties

#### 2.3.1 Surface tension and electrical conductivity

The surface tension of LPSG/PVA blends was measured at room temperature using the Wilhelmy plate method with a tensiometer (model Krüss® K100, Germany). The electrical conductivity of electrospinning solutions was measured using a digital conductivity meter (model 3540, Jenway, UK).

#### 2.3.2 Zeta potential

The zeta potential of LPSG/PVA solutions was measured by a zeta sizer (Zetasizer Nano, Malvern Instrument, UK). Samples were diluted with deionized water at a ratio of 1:50 (v/v) to determine the zeta potential (mV) of each dispersion.

#### 2.3.3 Viscosity

The viscosity and flow behavior parameters of LPSG/PVA solutions were determined using a Brookfield viscometer (159 DV III Ultra, Brookfield Engineering Laboratories, Stoughton, MA, USA) with a SC4-27 spindle at 25 °C and shear rates ranging from 1 to 85 s^−1^ (Komijani et al., 2022). The flow behavior of solusions was defined using the power law model (Eq. (1)) (Koocheki et al., 2013).

(1) $$\tau =k{\left(\dot{\gamma }\right)}^{n}$$

where, τ (Pa) is the shear stress, γ (s^−1^) is the shear rate, *k* (Pa.s^n^) is the power-law consistency coefficient and *n* is the dimensionless flow behavior index.

### 2.4 Electrospinning process

In order to prepare the electrospun nanofibers, 10 ml of the LPSG/PVA solution was loaded into a plastic syringe with an 18-gauge stainless steel needle (inner diameter of 0.838 mm and outer diameter of 1.270 mm, Sigma-Aldrich, USA) and placed in a mechanical syringe pump (NE- 1000, New Era Pump System Inc., Wantagh, NY, USA). The electrospinning process was carried out using the electrospinning unit (Electrospinning, Starter Kit-Yflow, Spain) with a fixed flow rate of 0.04 ml/h, applied voltage of 20 kV, and a needle tip-to-collector distance of 150 mm for 40 min at room temperature. A stainless-steel square plate with a dimension of 100 × 100 mm^2^ was used as the collector.

For encapsulation of D-limonene, the continues phase of emulsions was prepared by mixing 0.5 and 0.75% LPSG concentrations and PVA at a LPSG:PVA ratio of 30:70 (v/v), based on the preliminary study. Tween-20 (0.1% based on LPSG weight) was then added to the solutions. Following solution preparation, 30% D-limonene based on gum weight was added, and the mixture was then stirred for 5 min using a magnetic stirrer. The coarse emulsion was then homogenized for 3 min in a cold-water bath using an Ultra-Turrax homogenizer (model T-25, IKA Instruments, Germany) at a speed of 13000 rpm. To make sure the feed air bubbles disappeared, the sample was left at room temperature for 15 min. The emulsion was further electrospinned using the same method described above.

### 2.5 Morphology of LPSG/PVA nanofibers

The Scanning Electron Microscope (SEM) (Model VP-1450, LEO Co, Germany) was used at an increasing voltage of 20 kV to define the LPSG/PVA electrospun nanofibers morphology. The nanofiber diameters were calculated using image visualization software ImageJ (ImageJ, National Institutes of Health). About 100 fibers from SEM images were randomly selected to measure the mean nanofiber diameters (Charpashlo et al., 2021).

### 2.6 Fourier transform infrared spectroscopy (FTIR)

The structure and possible interactions between the components of the nanofibers and the core material (D-limonene) were studied using FTIR analysis (Zhang et al., 2019). The FTIR analysis was carried out at room temperature using Infrared Fourier Transmission Spectroscopy (FTIR, AVATAR 370, Thermo Nico-let, USA) in the wave numbers ranging from 400 to 4000 cm^−1^.

### 2.7 Differential scanning calorimetry (DSC)

The thermal properties of LPSG powder, PVA powder, D-limonene, and produced nanofibers with and without D-limonene were measured using a differential scanning calorimeter (DSC- 100, Spico Co., Tehran, Iran). The thermograms were recorded by increasing the temperature from 25 to 400 °C, with a heating rate of 10 °C/min (Zarekhalili et al., 2017).

### 2.8 Encapsulation efficiency (EE)

Initially, 1 mg of loaded nanofibers and 2 ml of ethanol with a purity of 99% were mixed for 10 min in a tube shaker to remove the untrapped D-limonene on the surface of the nanofibers. To separate the LPSG/PVA nanofibers and D-limonene, a microfilter was used (filter pore size 0.45 μm, Amicon, Merck Company, Germany). The amount of D-limonene was then measured at 252 nm using a UV–VIS spectrophotometer (WPA, S2000UV/Vis, England) (Evageliou and Saliari, 2017; Tayebi-Moghaddam et al., 2021). The encapsulation efficiency (EE), loading capacity (LC), surface D-limonene (SD), and encapsulation yield (EY) were calculated using equations (2), (3), (4), (5)) (Khoshakhlagh et al., 2017).

(2) $$\text{EE}\%=\frac{W1}{W2}\times 100$$

where, w_1_ is the measured weight of D-limonene in the certain weight of nanofibers, and w_2_ is the used weight of D-limonene for production the same amount of LPSG/PVA nanofibers.

(3) $$\text{LC}\%=\frac{\;w1-W3}{W4}\times 100$$

where, w_3_ is the amount of free D-limonene in the filtered ethanol for a given weight of nanofibers and w_4_ is the weight of nanofibers.

(4) $$\text{SD}=\frac{W3}{W1}\times 100$$

(5) $$\text{EY}=\frac{W4}{W5}\times 100$$

where, w_5_ is the weight of solid mass in the electrospinning solutions.

### 2.9 *In vitro* release kinetics of D-limonene

The D-limonene release kinetics were measured in accordance with Commission Regulation October 2011 EU (10/2011/EC) using different simulated food media, including distilled water, acetic acid 3%, ethanol 50%, and ethanol 10%, which are considered as food simulants for aqueous, acidic, oily, and alcoholic or alkaline food products, respectively (Atay et al., 2018).

To measure the release of D-limonene, 5 mg of D-limonene-loaded nanofibers were added to a dialysis bag (nominal 12 kDa, Sigma-Aldrich) containing 3 ml of the food simulant (Rezaeinia et al., 2020b). The dialysis bags were then floated in 50 ml of each simulated food media and stirred at 100 rpm for 10 min at ambient temperature. Every 5 s for the first 2 min and then 30 as the end of the process (150 s), 3 ml of samples were taken, mixed with 5 ml of ethanol, centrifuged for 10 min at 5000 rpm, and their absorbance values were calculated using a UV–visible spectrophotometer at 252 nm.

The mechanisms underlying the release of D-limonene from the nanofibers were examined using a number of kinetic models, including Kopcha (Eq. (6)), Peppas-Sahlin (Eq. (7)), Rigter-Peppas (Eq. (8)), and Higuchi (Eq. (9)).

(6) $$M_{t}=A\times t^{0.5}+B\times t$$

(7) $$\frac{M_{t}}{M_{\infty }}={k_{1}t}^{m}+{k_{2}t}^{2m}$$

(8) $$\frac{M_{t}}{M_{\infty }}=k^{n}$$

(9) $$\frac{M_{t}}{M_{\infty }}={k\times t}^{1/2}$$

where, M_t_ is the amount of D-limonene released at time *t*, M_∞_ is the initial weight of D-limonene loaded in nanofibers. A and B are diffusion and erosion rate constants. The A/B ratio defines the release mechanism in the Kopcha model; A/B > 1 is diffusion, A/B < 1 shows the erosion, and if the A/B = 1, both the diffusion and erosion mechanisms are happening (Kopcha et al., 1990). *k*_*1*_ and *k*_*2*_ are the Fickian and erosion rate index, respectively. *m* is the diffusion exponent (Eq. (7)). *k*_*1*_>*k*_*2*_ confirms the Fickian-diffusion release, *k*_*2*_>*k*_*1*_, describes the erosion, and if the ratio is equal to 1, erosion and diffusion mechanisms are both effective (Peppas and Sahlin, 1989). The *n* exponent in the Rigter-Peppas model is used to determine the release kinetics (Eq. (8)) and when *n* ≤ 0.45, the dominant mechanism is Fickian-diffusion, 0.89 ≤ *n* ≤ 1 expressed the erosion mechanism, whereas 0.45 <*n* < 0.89 are governed by non-Fickian mechanism (Peppas and Sahlin, 1989). In Eq. (9) *k* is the release constant and exposes the erosion mechanism (Higuchi, 1963).

### 2.10 Statistical analysis

All of the measurements were done in triplicate. IBM SPSS statistics software (version 22.0, IBM Corp., USA), was applied to obtain data based on the completely randomized design with the factorial arrangement. Duncan's test was conducted with the statistical significance level of P < 0.05.

## 3 Results and discussion

### 3.1 Physicochemical properties of polymer solutions

#### 3.1.1 Surface tension

The surface tension of deionized water was about 73.55 mN/m. Due to the presence of protein in the LPSG (Soleimanpour et al., 2013a, 2013b), the surface tension significantly decreased with the addition of gum and increase in its concentration from 0.25% (w/v) to 1% (w/v) (Table 1). Acacia gum also reduced the surface tension of deionized water from 62.27 (mN/m) to 51.29 (mN/m) (Zaeim et al., 2018) which is almost similar to the data of the present study.

**Table 1:** Effects of LPSG concentration and LPSG to PVA ratio on the surface tension, electrical conductivity, and zeta potential of solution.

LPSG (%)	LPSG:PVA (v/v)	Surface tension (mN/m)	Electrical conductivity (μ/s)	Zeta potential (mv)	Power law parameters		
					*n*	*k* (mPa.s)	R^2^
**0.25**	**100:0**	63.66 ± 0.11^a^	331.12 ± 2.18^k^	−37.43 ± 4.06^d^	0.48^d^	2.38^g^	0.991
**0.5**		60.07 ± 0.04^b^	373.67 ± 3.12^i^	−41.22 ± 3.85^c^	0.45^d^	2.87^g^	0.997
**0.75**		58.81 ± 0.10^c^	515.62 ± 4.30^f^	−47.92 ± 16.01^b^	0.42^d^	4.33^fg^	0.996
**1**		54.33 ± 0.03^d^	1211.37 ± 6.66^a^	−52.83 ± 7.68^a^	0.41^d^	17.20^a^	0.994
**0.25**	**90:10**	51.48 ± 0.01^e^	313.67 ± 4.05^lm^	−16.30 ± 7.01^h^	0.50^cd^	2.07^g^	0.992
**0.5**		49.25 ± 0.03^f^	354.10 ± 3.02^j^	−21.57 ± 5.2^fg^	0.49^d^	2.46^g^	0.992
**0.75**		47.86 ± 0.07^g^	487.57 ± 3.12^g^	−22.64 ± 9.14^f^	0.46^d^	3.95^fg^	0.994
**1**		45.83 ± 0.03^gh^	1049.67 ± 1.53^b^	−24.83 ± 5.2^e^	0.45^d^	14.7^b^	0.997
**0.25**	**70:30**	50.27 ± 0.03^ef^	301.33 ± 3.10^mn^	−14.95 ± 18.3^i^	0.59^cd^	1.97^h^	0.994
**0.5**		48.73 ± 0.01^f^	328.22 ± 2.14^l^	−17.31 ± 17.8^gh^	0.57^cd^	2.15^g^	0.991
**0.75**		46.21 ± 0.02^g^	423.66 ± 2.21^h^	−20.70 ± 5.6^g^	0.56^cd^	2.63^g^	0.992
**1**		45.52 ± 0.13^gh^	927.67 ± 4.16^c^	−22.41 ± 1.3^f^	0.53^cd^	11.64^c^	0.992
**0.25**	**50:50**	49.22 ± 0.03^f^	283.2 ± 6.20^o^	−14.53 ± 12.7^i^	0.72^bc^	1.21^h^	0.993
**0.5**		47.12 ± 0.02^g^	314.35 ± 2.13^mn^	−15.12 ± 7.14^hi^	0.67^c^	1.69^h^	0.993
**0.75**		45.20 ± 0.01^gh^	374.51 ± 3.15^i^	−19.10 ± 10.5^g^	0.63^c^	2.21^g^	0.993
**1**		44.50 ± 0.01^h^	790.56 ± 2.54^d^	−20.24 ± 2.7^g^	0.60^c^	10.02^d^	0.991
**0.25**	**30:70**	47.12 ± 0.04^g^	275.0 ± 1.60^op^	−13.64 ± 7.5^i^	0.82^b^	1.05^h^	0.992
**0.5**		45.67 ± 0.03^gh^	288.61 ± 2.40^no^	−14.48 ± 19.2^i^	0.78^bc^	1.36^h^	0.995
**0.75**		43.19 ± 0.60^h^	323.16 ± 2.64^lm^	−16.2 ± 8.3^h^	0.75^bc^	1.92^gh^	0.992
**1**		43.39 ± 0.11^h^	696.33 ± 2.51^e^	−17.48 ± 4.6^gh^	0.74^bc^	7.40^e^	0.993
**0.25**	**10:90**	46.55 ± 0.01 ^g^	264.68 ± 3.02^p^	−12.35 ± 4.9^i^	0.87^b^	5.01^f^	0.991
**0.5**		44.37 ± 0.14^h^	267.51 ± 43.20^p^	−13.16 ± 4.90^i^	0.84^b^	1.13^h^	0.993
**0.75**		42.29 ± 0.20^i^	286.17 ± 3.07^o^	−14.82 ± 13.5^i^	0.81^b^	1.02^h^	0.995
**1**		42.61 ± 0.20^i^	514.27 ± 0.81^f^	−15 ± 12.3^hi^	0.80^b^	0.93^i^	0.992
**PVA**	**0:100**	41.26 ± 0.01^i^	260.68 ± 2.03^p^	−11.12 ± 8.3^j^	1.04^a^	0.89^i^	_

Different letters in each column show significant differences (*p* < 0.05).

Among all solutions, PVA had the lowest surface tension (Table 1). As a result, the surface tension of solutions decreased as the PVA content in the LPSG/PVA blend increased. Formation of intra-and inter-molecular hydrogen bonds between the hydroxyl groups of PVA and the carbonyl groups of gum could be the reason for increase in the surface activity of LPSG/PVA blend (Padil and Černík, 2015). According to Padil and Černík (2015) addition of PVA, and a reduction in surface tension caused the solvent molecules to diffuse among the karaya gum molecules, preventing the formation of beads. Previous research on Balangu seed gum and PVA, also showed that the surface tension decreased as the ratio of PVA in the blend increased (Rezaeinia et al., 2020a).

The solution drop is suspended from the needle tip by molecular abduction and surface tension (Kriegel et al., 2009). When voltage is applied, electric charges are transferred to the surface of the suspended droplet, causing the margins of the solution surface to briefly oscillate and the droplet to change shape into a cone (Lan et al., 2019). The polymer solution erupts as a charged jet when the voltage is increased above the critical point, producing shear stresses that are strong enough to break through the surface tension of the solution (Long et al., 2019). When a high voltage is applied, the jet becomes unstable (Kriegel et al., 2009).

Since solvent molecules cannot diffuse among polymer molecules in high surface tension solutions, they tend to aggregate and form spherical shapes, which leads to the formation of beads in the fibers (Kriegel et al., 2009; Spasova et al., 2006). As a result, using PVA as a co-spinning polymer is helpful in the reduction of solution surface tension.

#### 3.1.2 Electrical conductivity

The electrical conductivity increased with increasing LPSG concentration and decreased with addition of PVA and increasing its volume ratio in the mixture (Table 1). According to Santos et al. (2014), the electrical conductivity of a solution is directly proportional to the number of charges present. Since LPSG is an anionic polymer (Soleimanpour et al., 2013b), the increase in its concentration led to an increase in the negative electrical charges and the solution's conductivity. LPSG is a desirable biopolymer for electrospinning because of its high electrical conductivity and capacity to elongate the jet, which were helpful for the electrospinning procedure and the production of homogeneous fibers (Zong et al., 2002).

Due to PVA's weaker anionic nature, the electrical conductivity of the polymer solution decreases with the addition of PVA and increasing its content in the mixture (Golkar et al., 2019). Kurd et al. (2017) also discovered that the electrical conductivity of the solution decreased as the ratio of PVA to basil seed gum increased.

The structure of hydrocolloids in an aqueous solution significantly affects the electrical conductivity (Zaeim et al., 2018), as this physical parameter depends on the interactions between water molecules and charged carboxyl or hydroxyl groups (Morales-Sánchez et al., 2015). Solutions with low viscosities encourage ion mobility, which improves the conductivity (de Diego, 2001). The solution conductivity and the applied electrostatic field both have impacts on the net charge density carried by the erupting jet (Ghorani et al., 2017; Zhao et al., 2004). The ability to transfer charge to the jet also increases as the number of ions in the solution increases. As a result, the jet's elongation increases, and the average diameter of the created fibers decreases (Ghorani et al., 2017; Zhang et al., 2018, Zhang et al., 2018).

#### 3.1.3 Zeta potential

When gum concentration increased, the zeta potential decreased due to the presence of high acidic fractions in LPSG structure (Soleimanpour et al., 2013b). Zeta potential decreased by the addition of PVA to the mixture (Table 1). Due to the presence of acetate groups in its structure, PVA has a negative electric charge (Wiśniewska, 2011). According to Brunchi et al. (2016), the decrease in the mixed solution's zeta potential results from a reduction in the gum's side chain mobility when the PVA proportion is increased in the gum/PVA solution. They also showed that fibers with high beads were formed when solutions with higher zeta potential were used. Formation of beads could be attributed to the direct impact of the zeta potential on the solution's electrical conductivity (Rezaeinia et al., 2020a). A decrease in the electrical conductivity results in a reduction in the average diameter of fibers (Santos et al., 2014). When the electrical conductivity of the solution is too high, the polymer jet becomes unstable and beads begin to accumulate in the fibers (Angammana and Jayaram, 2011; Hemamalini and Giri Dev, 2018).

Similar to our findings, when the *Lallemantia royleana* seed gum dissolved in distilled water, the accessibility of conductive ionic components increased (Rezaeinia et al., 2020a). Additionally, because of the gum's negative zeta potential, a weak electrolyte was created, and the solution's electrical conductivity increased.

#### 3.1.4 Viscosity

**Fig. 1:**
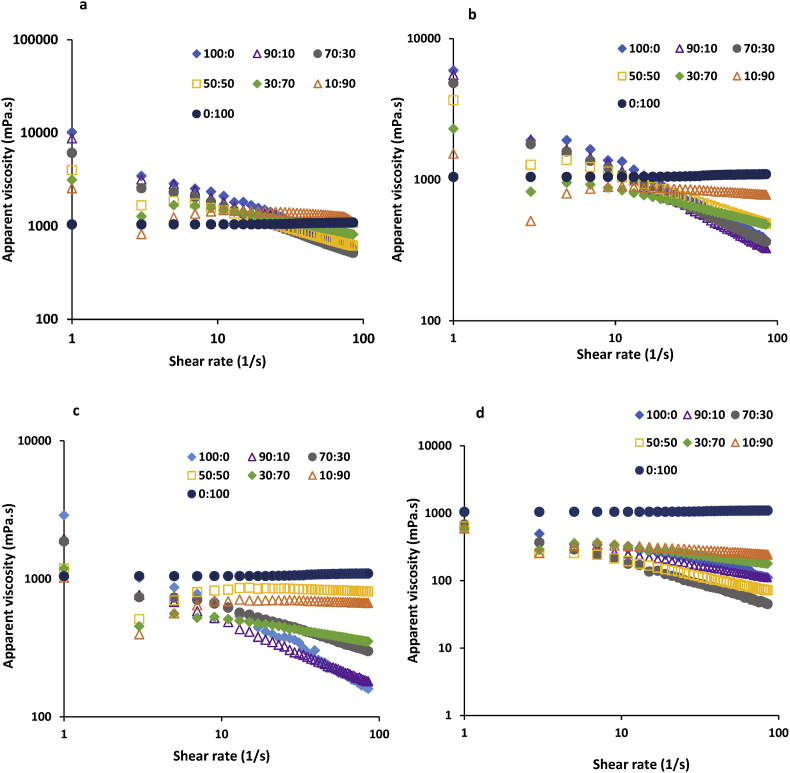
Apparent viscosity of solutions with different LPSG: PVA mixing ratios (100:0, 90:10, 70:30, 50:50, 30:70, 10:90, and 0:100 v/v) and different LPSG concentrations (a) 1%, b) 0.75% c) 0.5% and d) 0.25%).

Among all samples, the 1% (w/v) LPSG solution had the highest viscosity (Fig. 1). All LPSG/PVA mixed solutions had pseudoplastic behavior, with viscosity decreasing as shear rate increased. According to Koocheki et al. (2022), this occurs because the shear force disrupts the links between polymer chains, causing the molecules to align to the direction of the shear force. The viscosity and consistency coefficient (*k*) of solutions increased while the flow behavior index (*n*) decreased as LPSG concentrations and their proportion in the LPSG/PVA mixture increased (Table 1). The greater consistency coefficient is expected to be caused by an increase in the intermolecular contacts and polymer chain entanglements (Koocheki et al., 2013). Additionally, a higher gum content increases the hydrophilic groups in the solution, which improves the ability to absorb more water via hydrogen bonding. One of the reasons why beads appear in the fibers during the electrospinning process is the solution's low viscosity (Zhang et al., 2018, Zhang et al., 2018). However, transferring the polymer solution from the needle to the collector is challenging when the viscosity of solution is high (Sill and von Recum, 2008). Therefore, it is crucial to find a solution viscosity that allows the polymer to spin easily and prevents beads from forming in the nanofibers. Shear thinning behavior is a limiting factor in the electrospinning process because the high shearing force at the tip of the electrospinning needle decreases the viscosity and prevents the formation of Taylor cone jet. The gum's pseudoplastic behavior reduced after the addition of PVA, which lowered the gum's shear thinning behavior when the shear force was applied at the needle's tip (Rezaeinia et al., 2019). Therefore, PVA added to the gum solution helped the formation of a stable jet by reducing the shear thinning behavior at the tip of the needle.

### 3.2 Nanofibers analysis

#### 3.2.1 Nanofibers morphology

Spinnability and jet formation are greatly influenced by solution surface tension, electrical conductivity, polymer concentration, and viscosity (Ghorani and Tucker, 2015). Formation of jet was prevented, and even the syringe was blocked by the high viscosity of 1% (w/v) LPSG. Extreme viscosity is confirmed to be a limiting factor which makes the electrospinning process difficult (Shenoy et al., 2005). In addition, since 0.25% (w/v) LPSG had the lowest electrical conductivity and highest surface tension, spherical shapes and bead defects developed, and the jet became unstable. Therefore, these concentrations were not suitable for the formation of nanofibers.

**Fig. 2:**
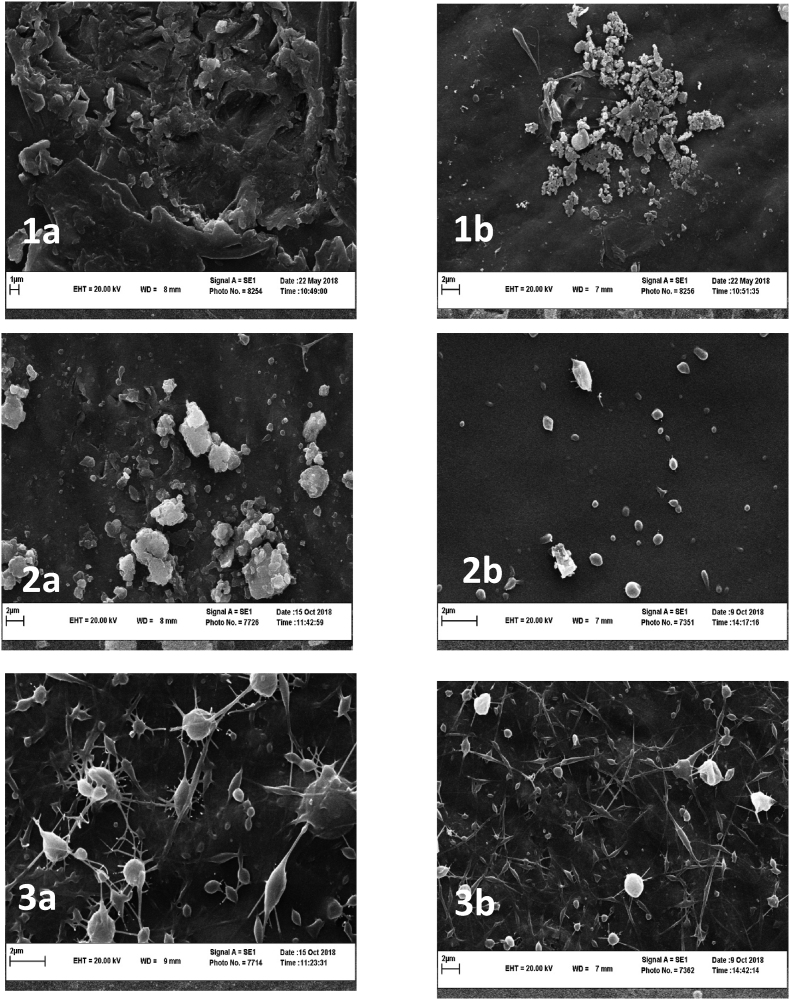

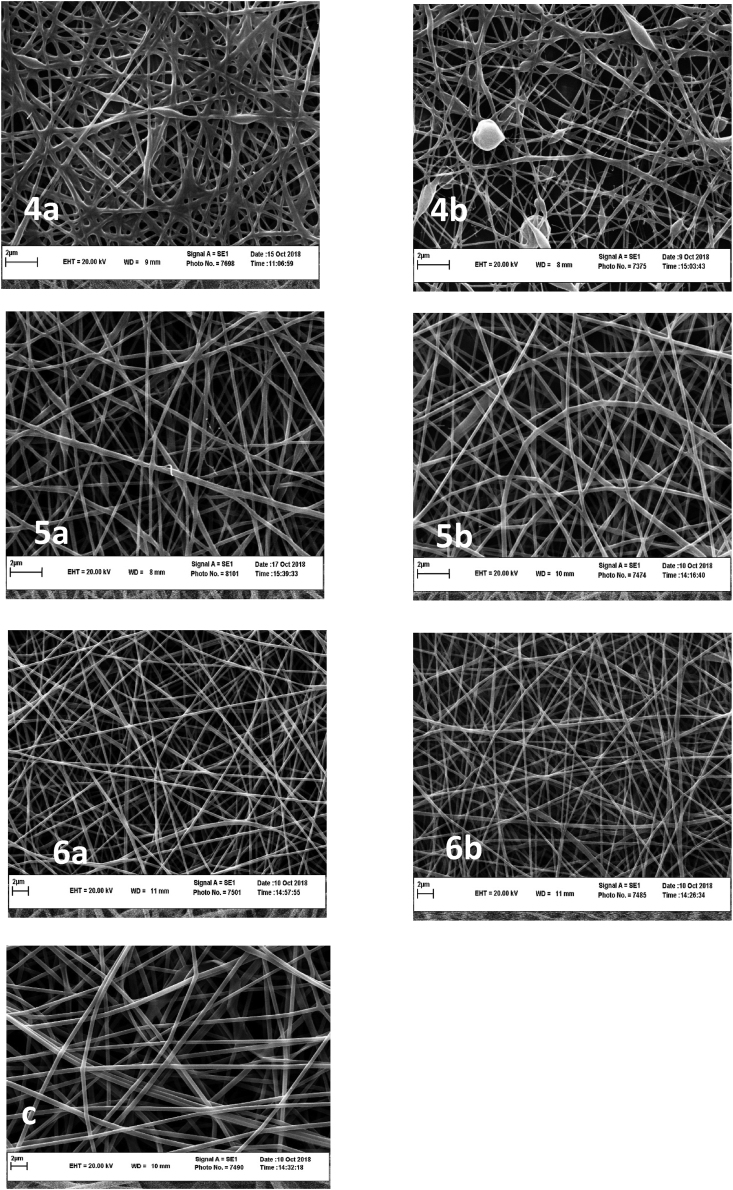
SEM images of nanofibers from LPSG/PVA nanofibers. a) 0.5% and b) 0.75% LPSG, and c) pure PVA; LPSG to PVA volume ratios of 1) 100: 0, 2) 90:10, 3) 70:30, 4) 50:50, 5) 30:70 and 6) 10:90.

At LPSG concentrations of 0.75% and 0.5% (w/v) for volume ratios of 100:0 and 90:10 LPSG: PVA, only sprayed particles were generated (Fig. 2, and Table 2). The electrospinning process and the formation of nanofibers from LPSG are difficult due to the high viscosity of the polymer (Gómez-Mascaraque et al., 2017). Therefore, adding PVA is necessary to overcome this drawback. The beads and spindle-like beads formed and tiny fibers emerged at 70:30 LPSG to PVA volume ratio, whereas at 50:50 volume ratio, nanofibers with web-like patterns appeared at both LPSG concentrations. As the content of PVA increased from 30:70 to 10:90 LPSG to PVA volume ratios, homogeneous fibers were formed. Aadil et al. (2019) also reported that decreasing the surface tension by increasing the PVA to gellan gum ratio resulted in a decrease in bead production because of the impact of low surface tension on jet. According to Padil and Černík (2015), due to an increase in the electrical conductivity and viscosity, adding more gum to the gum/PVA mixture inhibits the formation of continuous and uniform fibers.

**Table 2:** Nanofibers’ morphology and average diameters of nanofibers from 0.5 to 0.75% LPSG with different LPSG/PVA ratios.

LPSG (%)	LPSG/PVA (v/v) ratios	Morphology	Nanofiber diameters
**0.5**	**100:0**	Beads	_
	**90:10**	Beads	_
	**70:30**	Beads with small fibers	126.86 ± 44.693
	**50:50**	Fibers with beads	150.85 ± 30.087
	**30:70**	Nanofibers with no beads	190.94 ± 36.438
	**10:90**	Nanofibers with no beads	273.58 ± 51.774
**0.75**	**100:0**	Beads	_
	**90:10**	Beads	_
	**70:30**	Beads with small fibers	98.86 ± 37.804
	**50:50**	Fibers with beads	106.36 ± 27.297
	**30:70**	Nanofibers with no beads	174.22 ± 27.848
	**10:90**	Nanofibers with no beads	222.31 ± 53.60
**Pure PVA**	**0:100**	Nanofibers with no beads	695.95 ± 124.34
**Loaded nanofibers (0.5% LPSG)**	**30:70**	Nanofibers with no beads	208.55 ± 41.282
**Loaded nanofibers (0.75% LPSG)**	**30:70**	Nanofibers with no beads	192.17 ± 62.015

With increasing LPSG to PVA ratios at both 0.5 and 0.75% gum concentrations, the fiber diameter decreased (Table 2). At all LPSG to PVA mixing ratios, the diameter of the fibers obtained from 0.75% gum concentration was smaller than that of 0.5% gum concentration. Higher electrical conductivity, which results in more repulsion force at the jet surface, along with increased polymer chain entanglement and solution viscosity, result in an elongated polymer jet in the electrical field and thinner nanofiber production (Rošic et al., 2012; Uyar and Besenbacher, 2008). Kurd et al. (2017) and Rezaeinia et al. (2020) also concluded that by increasing the balangu gum and basil seed gum concentrations and their ratio in gum/PVA mixtures, thinner fibers were produced, due to an increase in the viscosity and electrical conductivity of polymer solutions.

**Fig. 3:**
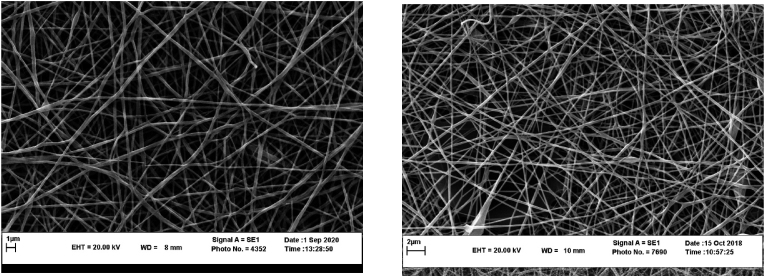
SEM images of LPSG/PVA nanofibers containing 3% (w/w) D-limonene. left: 0.5%, and right: 0.75% LPSG.

Therefore, the research was continued using the ratio of 30:70 (v/v) LPSG:PVA to prepare nanofibers for the encapsulation of D-limonene. The homogeneity, uniformity, and bead-free nanofibers were not affected by D-limonene after its encapsulation within LPSG/PVA nanofibers (Fig. 3). The diameter of nanofibers with 0.5% LPSG concentration was larger than that of nanofibers with 0.75% concentration, even after D-limonene encapsulation. The results showed that, in comparison to nanofibers without D-limonene, the diameter of LPSG/PVA nanofibers containing D-limonene at both 0.5% and 0.75% LPSG concentrations was higher. Kurd et al. (2019), who studied the encapsulation of hespertin in basil seed gum/PVA fibers, came to a similar conclusion regarding the increase in fiber diameter following the addition of essential oil.

#### 3.2.2 FTIR spectroscopy

**Fig. 4:**
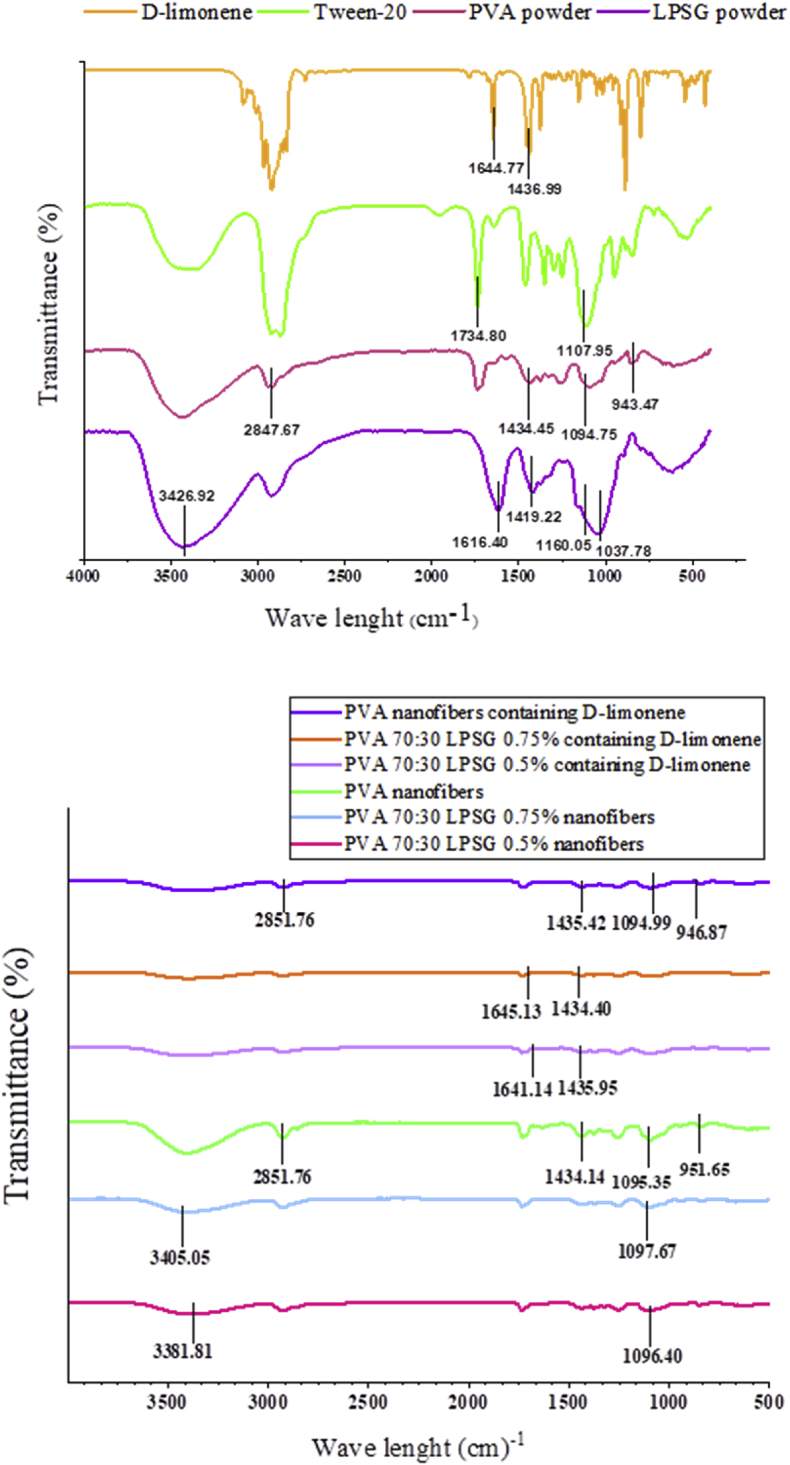
FTIR spectrums of LPSG powder, PVA powder, D-limonene, PVA fibers, LPSG:PVA (30:70 v/v) nanofibers and D-limonene incorporated LPSG/PVA nanofibers.

FTIR analysis can be used to determine the structures and functional groups of polymers, validate the compatibility of blended polymers, and confirm incorporating agents (Monjazeb Marvdashti et al., 2017; Zeighampour et al., 2018). Fig. 4 provides more details on the FTIR spectra of LPSG and PVA powders, D-limonene, and tween 20. The stretching vibration of the hydroxyl groups in LPSG powder generated a broad peak in the 3700–3000 cm^−1^ region of the FTIR spectrum, particularly at 3426.92 cm^−1^. This peak is associated with the water absorption of LPSG (Khoshakhlagh et al., 2017). The peak between 2500 and 3000 cm^−1^ is associated with C- H stretching vibration (Yuen et al., 2009). The peaks at 1616.40 cm^−1^ and 1419.22 cm^−1^ are caused by the stretching vibrations of C−O of amide I and the symmetric COO, indicating the presence of a small amount of peptide in LPSG (Ebrahimi et al., 2016). The symmetric stretching bands of COO could be related to the presence of uronic acids in LPSG structure (Koocheki et al., 2022). Carbohydrate fingerprints can be found in the region between 750 and 1300 cm^−1^ (Şen and Erboz, 2010). The absorption at 1037.78 cm^−1^ is associated with the fingerprint of LPSG. The peaks in this region are caused by carbohydrates C−O ether groups and the vibrational C−O−C of the glycosidic bond between monosaccharide units (Khoshakhlagh et al., 2017).

The intermolecular and intramolecular hydrogen bonds within the hydroxyl groups are responsible for the observed peaks in the PVA powder spectrum between 3200 and 3500 cm^−1^ (Hosseini et al., 2021). The characteristic peak at 1094.75 cm^−1^ for PVA is associated with the stretching vibrations in the crystalline polymer's C−OH groups brought on by the intramolecular hydrogen bond between two OH groups, and the peak at 1262.09 cm^−1^ is related to the bending CH_2_ (Fahami and Fathi, 2018; Kurd et al., 2017). In the spectra of PVA nanofibers, characteristic peaks observed in the PVA powder, including those at 853 cm^−1^, 943 cm^−1^, 1094 cm^−1^, and 1376 cm-1, were also present. However, the higher intensities and slight shifts in wave numbers from 2847.67 cm^−1^ to 2851.76 cm^−1^, 1434.45 cm^−1^ to 1434.14 cm^−1^, 1094.75 cm^−1^ to 1095.35 cm^−1^, and 943.47 cm^−1^ to 951.65 cm^−1^ indicated an increase in the crystallinity of PVA nanofibers compared to its powder form (Rajeswari et al., 2013; Subramanian et al., 2014). The peaks associated with C−O stretching vibrations in C−OH groups are believed to be connected to the crystalline regions of polymers (Zeighampour et al., 2018).

The shifts of the wavenumbers in the 3000–3500 cm^−1^ region towards the lower frequencies indicate the formation of hydrogen bonds between the hydroxyl groups of LPSG and PVA (Monjazeb Marvdashti et al., 2017; Zhang et al., 2019). Following the production of LPSG/PVA nanofibers, the peaks corresponding to LPSG at 3426.92 cm^−1^ shifted to 3381.81 cm^−1^ for 0.5% LPSG (w/v) and 3405.05 cm^−1^ for 0.75% LPSG (w/v).

These shifts result from the stretching vibration of OH in the infrared spectrum of the nanofibers, indicating the miscibility of the LPSG and PVA, along with the hydrogen bonds that have formed between them. Similar results have been reported regarding the miscibility of *Alyssum homolocarpum* seed gum and PVA (Monjazeb Marvdashti et al., 2017). The peaks at 1160.05 cm^−1^ in LPSG powder and 1094.75 cm^−1^ in PVA spectra shifted to 1097.67 cm^−1^ for 0.75% LPSG and 1096.40 cm^−1^ for 0.5% LPSG in LPSG/PVA nanofiber's spectrum. According to Zhang et al. (2019), these shifts are caused by the interactions between the primary and secondary alcohol groups through hydrogen bonds in the blends of xanthan gum and PVA.

The characteristic peaks at 1436.99 cm^−1^ and 1644.77 cm^−1^ for D-limonene, were selected to track its encapsulation within the LPSG/PVA nanofibers. These peaks shifted to 1435.95 cm^−1^ and 1641.14 cm^−1^ for 0.5% LPSG, and 1434.40 cm^−1^ and 1645.13 for 0.75% LPSG in the LPSG/PVA nanofibers. These minor shift in the wave numbers proved that nanofibers effectively encapsulated D-limonene. Rezaeinia et al. (2019) also confirmed the encapsulation of *Mentha longifolia* L. essential oil within Balangu seed gum/PVA nanocapsules through observing the slight shifts in the characteristic peaks of the essential oil. It is important to note that the low concentration of D-limonene and Tween 20 in the fibers led to the combination of some of their minor peaks after loading, as a result of interference and overlap with peaks from other components.

#### 3.2.3 Differential scanning calorimetry analysis

The glass transition temperature (T_g_) for LPSG was 55.2 °C, which is higher than the room temperature (Table 3), indicating that gum has good thermal and structural stability in ambient conditions over time (Rocha-Selmi et al., 2013). Khoshakhlagh et al. (2017) reported similar results for *Alyssum homolocarpum* seed gum, showing a T_g_ above the ambient temperature, signifying thermal stability and structural preservation over time. An endothermic peak ranging from 77.9 to 103.1 °C, centered at 90.5 °C, corresponds to the melting point (T_m_) of LPSG powder, representing the melting point of its crystalline and semi-crystalline regions (Khoshakhlagh et al., 2017). Another endothermic peak at 243.1 °C results from the melting and disordering of the crystalline and semi-crystalline regions in LPSG. The final peak on the LPSG thermogram was as an exothermic peak between 313 °C and 356.2 °C, with a degradation enthalpy of 18.06 J/g, indicating LPSG high thermal stability (Jamshidi et al., 2020; Seyedi et al., 2014). Khoshakhlagh et al. (2017) also reported a similar exothermic peak for *Alyssum homolocarpum* seed gum in relation to its high thermal stability.

**Table 3:** DSC parameters for LPSG, PVA, D-limonene, LPSG/PVA (30:70) nanofibers.

Sample	Tg °C	Tm°C	Melting enthalpy J/g
LPSG powder	55.2	90.5	23.261
PVA powder	62.7	197.4	11.856
0.5 LPSG	58.31	171.5	39.388
0.75% LPSG	57.2	169.9	72.586
Loaded nanofibers (0.5% LPSG)		224.9	10.544
Loaded nanofibers (0.75% LPSG)		203.6	15.794
D-limonene		109.9	91.367

The T_g_ and T_m_ for PVA powder were 62.7 °C and 197.4 °C, respectively (Table 3), indicating a semi-crystalline structure for PVA (Wang and Hsieh, 2008). These values were similar to those reported by Padil and Černík (2015) and Rezaeinia et al. (2019). The melting temperatures of LPSG/PVA nanofibers at 0.5% and 0.75% LPSG concentrations were 169.9 °C and 171.5 °C, respectively. According to Pandit et al. (2019), as the amount of gum in gum arabic/PVA blend increased, the cross-linking between these two polymers reduced the intermolecular hydrogen bonding in PVA chains and decreased the crystallinity.

**Fig. 5:**
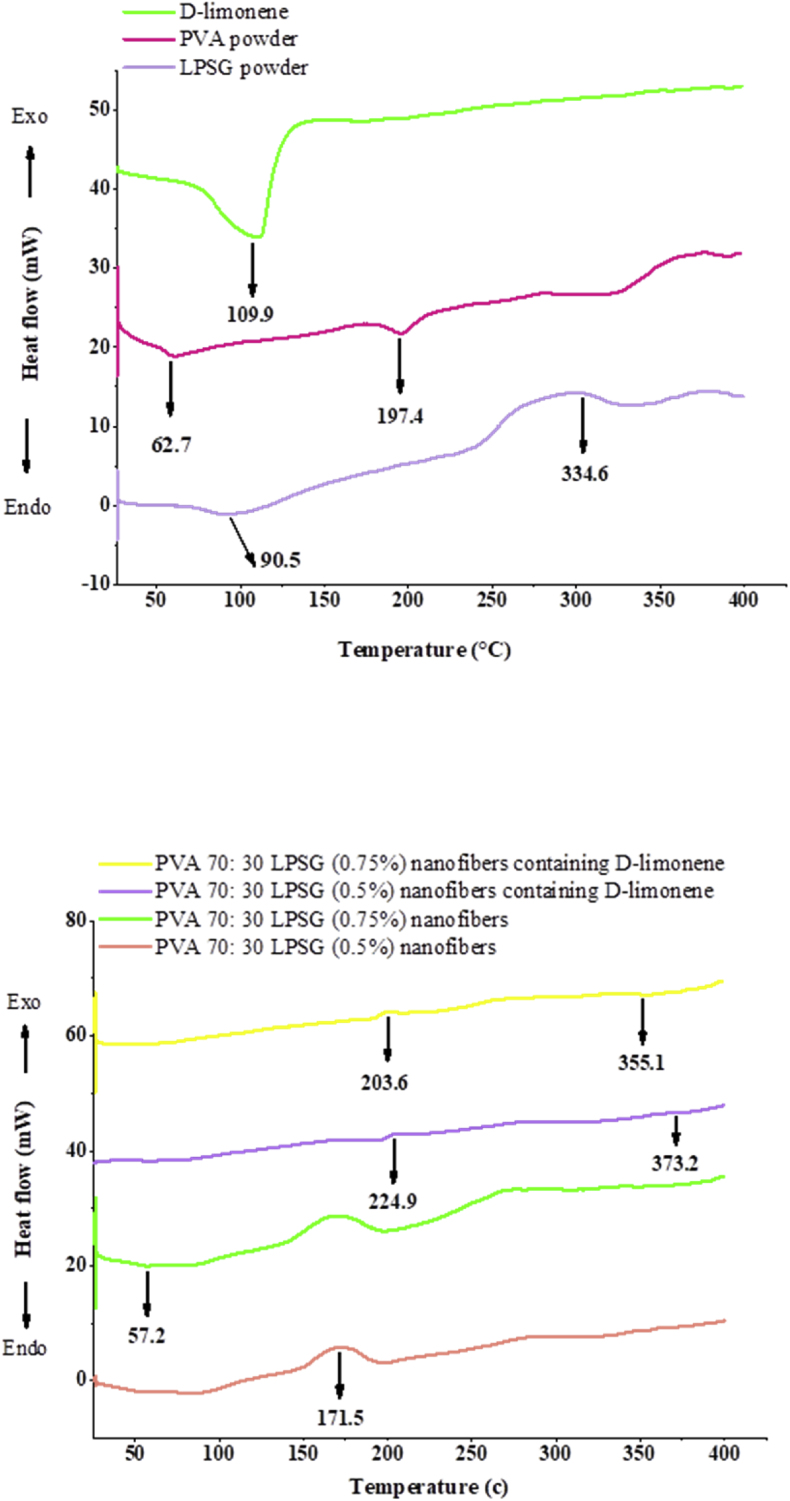
DSC thermograms of LPSG and PVA powders, nanofibers, and D-limonene before and after being incorporated into LPSG/PVA nanofibers.

The DSC thermogram of D-limonene revealed a sharp endothermic peak in the range of 82.4 °C–137.4 °C with its peak intensity at 109.9 °C, as a result of the evaporation of D-limonene (Fig. 5). Due to the heat sensitivity of D-limonene, the heat transfer rate increased steadily after 137.4 °C, confirming severe autoxidation and flavor degradation above this temperature. Alehosseini et al. (2021) and Khoshakhlagh et al. (2017) also reported similar D-limonene evaporation peaks and substantial degradation occurring above this critical temperature.

After D-limonene incorporation into LPSG/PVA nanofibers, the endothermic evaporation peak disappeared, implying that D-limonene was successfully entrapped in these nanofibers. Furthermore, there was no new peak visible in the aforementioned diagrams when compared to nanofibers and pure D-limonene, indicating that there was no interaction between the two substances. Similar to our results Rezaeinia et al. (2019) also concluded that the successful encapsulation of *Mentha longifolia* L. essential oil in *Lallemantia royleana* seed gum was demonstrated by the absence of the essential oil's evaporation peak and the lack of any interaction or appearance of new peak in the DSC thermogram of nanocapsules.

#### 3.2.4 Encapsulation efficiency (EE)

The encapsulation efficiency (EE) of D-limonene was higher for LPSG/PVA nanofibers than PVA alone, and it increased as the LPSG concentration increased (Table 4). The 0.75% (w/v) LPSG/PVA nanofibers also had the highest loading capacity and encapsulation yield, and the lowest surface D-limonene content. Formation of polysaccharide wall around D-limonene is the cause of the high encapsulation efficiency for LPSG/PVA fibers at both gum concentrations compared to pure PVA fibers (Díaz-Montes, 2023). According to Minemoto et al. (2002), the encapsulation efficiency increases as the ratio of wall material to core increases. The encapsulation efficiency is increased by the higher viscosity of 0.75% LPSG, which reduces the transfer of essential oil to the surface of the LPSG/PVA fibers during the electrospinning process (Carneiro et al., 2013). Khoshakhlagh et al. (2017) reached similar results regarding the extremely high encapsulation efficacy of D-limonene in electrosprayed *Alyssum homolocarpum* seed gum (EE% of 93.24%) compared to other encapsulation methods. D-limonene was more effectively encapsulated in 0.75% (w/v) LPSG/PVA (at mixing ratio of 30:70 v/v) electrospun nanofibers than it was in other materials, including spray-dried cassava starch (44%), chitosan-cellulose particles (51.29%), amylose nanostructures made by combining sonication and thermal processing (82%), and pectin-whey protein complexes (88%) (Galindo et al., 2020; Ganje et al., 2019; Ghasemi et al., 2018; Lopes et al., 2019; Ordoñez and Herrera, 2014).

**Table 4:** Encapsulation efficiency, loading capacity, surface D-limonene, and yield encapsulation of 0.5% and 0.75% (w/v) LPSG (30LPSG:70 PVA), and pure PVA nanofibers.

Nanofibers	Encapsulation efficiency (EE%)	Loading capacity (LC%)	Surface D-limonene (ppm)	Yield encapsulation (YE%)
**0.75**	96.23 ± 0.56^a^	2.80 ± 0.61^a^	81.50 ± 0.44^c^	92.13 ± 0.55^a^
**0. 5**	88.01 ± 0.46^b^	2.30 ± 0.57^a^	256.50 ± 0.74^b^	75.70 ± 0.39^b^
**pure PVA**	87.25 ± 0.43^b^	2.33 ± 0.60^a^	382.50 ± 0.64^a^	77.80 ± 0.42^b^

Different letters in each column show significant differences (*p* < 0.05).

#### 3.2.5 Release kinetics of D-limonene in the simulated food models

**Fig. 6:**
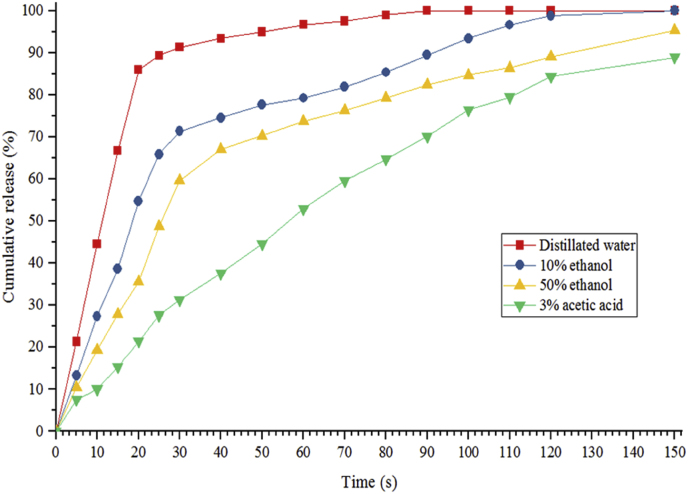
Release behavior of encapsulated D-limonene within LPSG/PVA nanofibers in simulated food models including liquid (distilled water), alcoholic or alkaline (ethanol 10%), oil (ethanol 50%), and acidic (acetic acid 3%).

The kinetics of D-limonene release in simulated foods, including liquid (distilled water), alcoholic or alkaline (ethanol 10%), oil (ethanol 50%), and acidic (acetic acid 3%), was investigated (Fig. 6). The 0.75% (w/v) LPSG/PVA nanofibers, which had the highest encapsulation efficiency and the smallest fiber diameter, were used to analyze the release kinetics of D-limonene essential oil. The highest amount of D-limonene was released in distilled water, and the least amount was in acetic acid. About 86% of the D-limonene was released from the LPSG/PVA nanofibers after 15 s of immersion in distilled water. This characteristic is explained by the hydrophilic nature of the biopolymers used for the encapsulation of D-limonene (Rezaeinia et al., 2019). Both LPSG and PVA are hydrophilic substances that form hydrogen bonds with water molecules when added to aqueous media like distilled water. While limonene release rate from LPSG/PVA nanofibers was significantly higher than that of zein-sodium caseinate microencapsules, it was comparable to that of whey protein isolate coated limonene during the burst release phase (Chen et al., 2018). Salehi et al. (2021) also reported that when limonene was encapsulated in β-cyclodextrin nanosponges, the highest gradient of limonene release was detected for water, followed by 50% and 10% ethanol media. Rezaeinia et al. (2020) investigated the mechanism of bergamot essential oil release from *Lallemantia royleana* seed gum/PVA nanofibers and found that it began rapidly and then progressed slowly due to the hydrophilic properties of the produced structures. Gum and PVA were less soluble in acetic acid than they were in distilled water (Rezaeinia et al., 2019), which led to a reduced release of D-limonene from the nanofibers.

To study the release mechanism of D-limonene from the LPSG/PVA nanofibers, the release kinetics results were fitted with a number of experimental models (Higuchi, Rigter-Peppas, Peppas-Sahlin and Kopcha). The coefficient of detemination (R^2^) and root mean square error (RMSE) indices were used to determine which model best described the release behavior of D-limonene (Alinaqi et al., 2021). The best model for describing the behavior of D-limonene release was found to be the Peppas-Sahlin model, which had the highest R^2^ and the lowest RMSE (Table 5).

**Table 5:** Models’ parameters of D-limonene release from LPSG/PVA (30:70) nanofibers in distilled water, ethanol 10% and 30%, and citric acid.

Model	Parameter	Distilled water	Ethanol 10%	Ethanol 50%	Acetic acid 3%
**Higuchi**	***k***	11.09	9.66	8.66	7.00
	**R**^**2**^	0.52	0.89	0.94	0.94
	**RMSE**	21.21	9.92	7.19	7.01
**Rigter-Peppas**	***k***	35.65	16.49	10.57	2.88
	**n**	0.23	0.38	0.44	0.37
	**R**^**2**^	0.85	0.94	0.95	0.99
	**RMSE**	12.2	7.89	7.13	3.11
**Peppas-sahlin**	***k***_***1***_	18.57	9.03	5.03	22.02
	***k***_***2***_	0.83	0.21	0.07	17.22
	**M**	0.54	0.63	0.73	0.22
	**R**^**2**^	0.94	0.97	0.98	0.99
	**RMSE**	8.09	5.88	4.79	2.37
**Kopcha**	**A**	21.31	13.62	10.37	3.89
	**B**	1.10	0.43	0.19	0.34
	**R**^**2**^	0.93	0.96	0.95	0.98
	**RMSE**	8.17	6.54	6.60	3.74

The *k*_*1*_/*k*_*2*_ index for the Peppas-Sahlin model was more than 1, the A/B index for the Kopcha model was greater than 1, and the *n* index for the Rigter-Peppas model was less than 0.45 in all solusions. As a result, the predominant efficient mechanism for the release of D-limonene in distilled water was type-I (Case-I), also known as the Fickian diffusion mechanism.

Due to the rapid dissolution of nanofibers in distilled water and the burst release of D-limonene from nanofibers (Fig. 5), these fibers could be used as a good fast dissolving carrier for beverages, oral care and health care products in pharmaceuticals and food technologies (Rezaeinia et al., 2019; Yildiz et al., 2019). Fast-dissolving nanofibers with electrohydrodynamic properties facilitate the dissolution of hydrophilic or less water-soluble substances, which is an advantageous property for drug delivery systems (Aytac et al., 2019; Yu et al., 2018).

## 4 Conclusion

In this study electrospinning technique was used to create uniform, small-diameter LPSG/PVA nanofibers for D-limonene encapsulation. The formation of nanofibers and the electrospinning process were made difficult by the high viscosity of the LPSG solution, which in certain cases even blocked the syringe and prevented the formation of the jet. Therefore, PVA was used, and a mixing volume ratio of 30 LPSG:70 PVA was selected to prepare nanofibers for the encapsulation of D-limonene. These nanofibers successfully entrapped D-limonene, and that there was no chemical reaction between the wall materials and D-limonene. The 0.75% LPSG/PVA nanofibers had the highest D-limonene encapsulation efficiency and the highest thermal stability. Release kinetics of D-limonene in simulated media followed a Fickian diffusion mechanism. The best model to explain the D-limonene release behavior from the LPSG/PVA nanofibers was the Peppas-Sahlin model. Since the amount D-limonene released in distilled water was higher than in other food models, these nanofibers could be used for fast-flavor release systems, such as beverages.

## CRediT authorship contribution statement

**Roya Kamalpour:** Formal analysis, Methodology, Writing – original draft. **Arash Koocheki:** Conceptualization, Supervision, Writing – review & editing. **Behrouz Ghorani:** Supervision, Methodology.

## Declaration of competing interest

The author declare that they have no conflict of interest.

## Data Availability

The authors do not have permission to share data.
